# Mechanism and isotherm modeling of effective adsorption of malachite green as endocrine disruptive dye using Acid Functionalized Maize Cob (AFMC)

**DOI:** 10.1038/s41598-021-00993-1

**Published:** 2021-11-02

**Authors:** John O Ojediran, Adewumi Oluwasogo Dada, Stephen O Aniyi, Robinson O. David, Adejoke D Adewumi

**Affiliations:** 1 Landmark University SDG 7 Research Group (Grow Affordable and Clean Energy), Omu-Aran, Nigeria; 2 Landmark University SDG 9 Research Group (Increase Industry, Innovation, and Infrastructure), Omu-Aran, Nigeria; 3 Department of Agricultural and Biosystems Engineering, Landmark University 9 (Increase Industry, Innovation, and Infrastructure), P.M.B.1001, Omu-Aran, Kwara, Nigeria; 4 Landmark University SDG 6 Research Group (Clean Water and Sanitation), Omu-Aran, Nigeria; 5 Landmark University SDG 11 Research Group (Sustainable Cities and Communities), Omu-Aran, Nigeria; 6 Industrial Chemistry Programme, Nanotechnology Laboratory, Department of Physical Sciences, Landmark University, P.M.B.1001, Omu-Aran, Kwara Nigeria; 7 Landmark University SDG GROUP 2 (Zero Hunger), Omu-Aran, Nigeria

**Keywords:** Environmental sciences, Chemistry, Materials science

## Abstract

Cationic Malachite green has been identified as a candidate for the endocrine disruptive compound found in the environment. In this study, the mechanism and isotherm modeling of effective adsorption of cationic malachite green dye onto acid-functionalized maize cob (AFMC) was investigated by batch technique. The operational parameters such as initial concentration (100–600 mg/L); contact time (10–120 min) and pH (3–10) influenced the removal efficiency and quantity adsorbed. A maximum of 99.3% removal efficiency was obtained at optimum conditions. AFMC physicochemical properties (surface area 1329 m^2^/g and particle size 300 μm < Ф < 250 μm) enhanced its efficiency. Based on R^2^ > 0.97 and consistently low values of adsorption statistical error functions (ASEF), equilibrium data were best fitted to Freundlich isotherm. Kinetic data were best described by a pseudo-second-order model with consistent R^2^ > 0.98 and validated by ASEF. The mechanism of the process was better described by intraparticle diffusion. Evidence of the adsorption process was confirmed by the change in morphology via Scanning Electron Microscopy (SEM) and surface chemistry by Fourier Transform infrared (FTIR). The performance of AFMC enlisted it as a sustainable and promising low-cost adsorbent from agro-residue for treatment of endocrine disruptive dye polluted water.

## Introduction

A healthy environment is a necessity for the actualization and realization of Sustainable Development Goals (SDG). However, the global release of Endocrine Disruptive Chemicals (EDC) through unregulated anthropogenic activities is troublesome. This Endocrine Disruptive Chemicals (EDC) have been identified as emerging contaminants that can hamper the hormonal system at little doses leading to the following hazardous effects: cancerous tumors, birth defects, and developmental disorders^1,2^. EDC can cause hormonal dysfunction, deficit brain disorder, body deformation, breast, prostate, and thyroid cancers, many cases of sexual development problems. The hazardous effects of EDC are not alien to some of the negative effects of some dyes and antibiotics released into the environment. malachite green dyes^3–7^.

The cationic dye of interest in this study is Malachite Green (MG) because of its versatility. It has found application in leather industries and in aquaculture as antiparasitic. It is used as a coloring agent in wool, silk, paper, etc.^8,9^. However, MG has been reported to be toxic, carcinogenic, and mutagenic. Malachite green could cause damage to humans and animals through direct inhalation and ingestion contact leading to various negative effects such as carcinogenesis, mutagenesis, teratogenesis, respiratory toxicity, and reduced fertility^10,11^. Systems and sensory organs of the body have been reported to be adversely affected by malachite green dyes^12,13^. MG does not biodegrade easily; it is known to be resilient to fading on exposure to light and water. More so, its removal from contaminated water via common conventional techniques (biological and chemical precipitation) is tough. However, its affinity for dissociation in solution makes it prone to liquid–solid adsorption. A cleaner environment and sustainable cities as part of the sustainable development goals would be difficult to achieve if the problem arising from endocrine disruptive chemicals are not combatted. Some of the explored methods of wastewater treatment are advanced oxidation, adsorption, photocatalytic degradation, and biodegradation^14,15^. Exceptional among this treatment technique is adsorption owing to its ease of operation, low cost, adaptation to a broad range of dyes, and design flexibility^16–18^. Adsorption of pollutant using biomass such as agrowaste materials have attracted attention of researchers. Biomass-based adsorbents are sustainable materials derived among others from agricultural residues, forest, animal manures, food processing wastes and municipal wastes and they have found relevance in adsorption studies^19,20^. They are low cost, readily available and researchers have leveraged on their functionalization and modification potencials in order to increase their efficiency. Numerous biomass have been used in adsorption studies. Among agrowaste and biomass reported to be efficient are Sugarcane bagasse^21^, biomaterial & chelating agent (Chitosan)^22^, Palm oil shell activated biomass^23,24^. Several sorbents have been reported effective in uptake of this EDC dye (MG Dye): *Ocimum gratissimum*^4^, magnetic biochar^25^, *Opuntia ficus-indica* activated carbon^26^, almond gum^9^, *Carica papaya* wood^27^, MOF nanocomposites^28^ Silico-manganese fume (SMF) waste^29^ para-aminobenzoic acid modified activated carbon^8^. Strength of selectivity and increase in the capacity of the sorbent with high removal efficiency could be enhanced by biomass functionalization. This has necessitated our interest in the modification of our sustainable low-cost agro-residue, maize cob. As a result, in this study, orthophosphoric acid has been used to functionalized and modified maize cob as low-cost agro-waste with the focus of achieving better sequestration. In this study, Acid Functionalized Maize Cob (AFMC) was developed purposely to effectively biosorb malachite green cationic dye as a candidate of endocrine disruptive chemical. Mechanistic and isotherm modeling of biosorption were explored. The statistical validity of the models using different error models was also investigated. Pre-and-post-adsorption characterization by surface morphology using Scanning Electron Microscopy (SEM) and surface chemistry by FTIR.

## Materials and methods

All chemicals used are of analytical grade. Orthophosphoric acid, H_3_PO_4_ (Loba Chemie), Hydrochloric acid, HCl (Loba Chemie CAS No: 7647-01-0, 37% purity), Sodium hydroxide, NaOH (Reckland Scientific Ec No: 215-185-5, 97% purity), Sodium Chloride, NaCl (Loba Chemie 99.5%, CAS: 7647-14-5), Malachite Green dye (CAS No.: 569-64-2; C_23_H_25_ClN_2_ (chloride); molar mass 364.911 g mol^−1^).

### Acid Functionalized Maize Cob (AFMC) as low-cost adsorbent

Purposive and simple random sampling technique which is the best time saving technique was used for the collection of the maize cob agro-waste from the dumpsite of the University being an Agricultural-based University. Maize cobs obtained from Landmark University (Agro-based University) were screened and cleaned thereafter dried at 105 °C for 5 h in Gen lab oven, crushed, grounded, and screened to 106 µm. Acid activation was carried out following the procedure in our previous study^30^ and elsewhere in other literature^31^ using 0.5 M ortho-phosphoric acid (H_3_PO_4_). A detailed typical procedure for the preparation of Acid Functionalized Maize Cob (AFMC) was explicitly presented in the supplementary material (SI).

### Physicochemical and spectroscopic characterization of AFMC

#### Determination of pH of AFMC

pH determination of AFMC was done by boiling 1 g AFMC in 100 mL distilled water for a period of 5 min. This was allowed to cool and its pH value was measured using an ATP-6 pH meter.

### Determination of AFMC bulk density

Weight difference divided by the volume as depicted in Archimedes’ principle was used for bulk density determination as depicted in Eq. (1)^32^

1 $$Bulk\;density = \frac{{w_{2} - w_{1} }}{v}$$

W_1_ = Weight of empty measuring cylinder, W_2_ = combination of AFMC mass and the crucible, V = volume.

#### Determination of AFMC moisture content

Moisture content was determined typically by introducing 5 g AFMC into the initially weighed crucible and heated for 1 h at 105 °C. Evaluation of the moisture content was done using Eq. (2)^42^

2 $$\% \;Moisture\;content = \frac{{w_{2} - w_{3} }}{{w_{2} - w_{1} }} \times 100$$

W_1_ = Weight of crucible, W_2_ = Initial weight of crucible with sample, W_3_ = Final weight of crucible with sample.

#### Determination of AFMC surface area by Saer’s method

The AFMC surface area was determined using Sear's method. This involves acidifying 0.5 g of each adsorbent with 0.1 M HCl to a pH of 3–3.5. The volume was made up to 50 mL with distilled water after the addition of 1 g of NaCl. The titration was carried out with standard 0.1 M NaOH at 298 K to pH 4, and then to pH 9.0 following the procedure reported in the literature^33,34^. The volume needed to raise the pH from 4 to 9 was noted and surface area evaluated using Eq. (3):

3 $${\text{S}}\left( {{\text{m}}^{{2}} /{\text{g}}} \right) \, = {\text{ 32V}} - {25}$$

### Batch biosorption studies

#### Preparation of malachite green adsorbate

**Figure 1:**
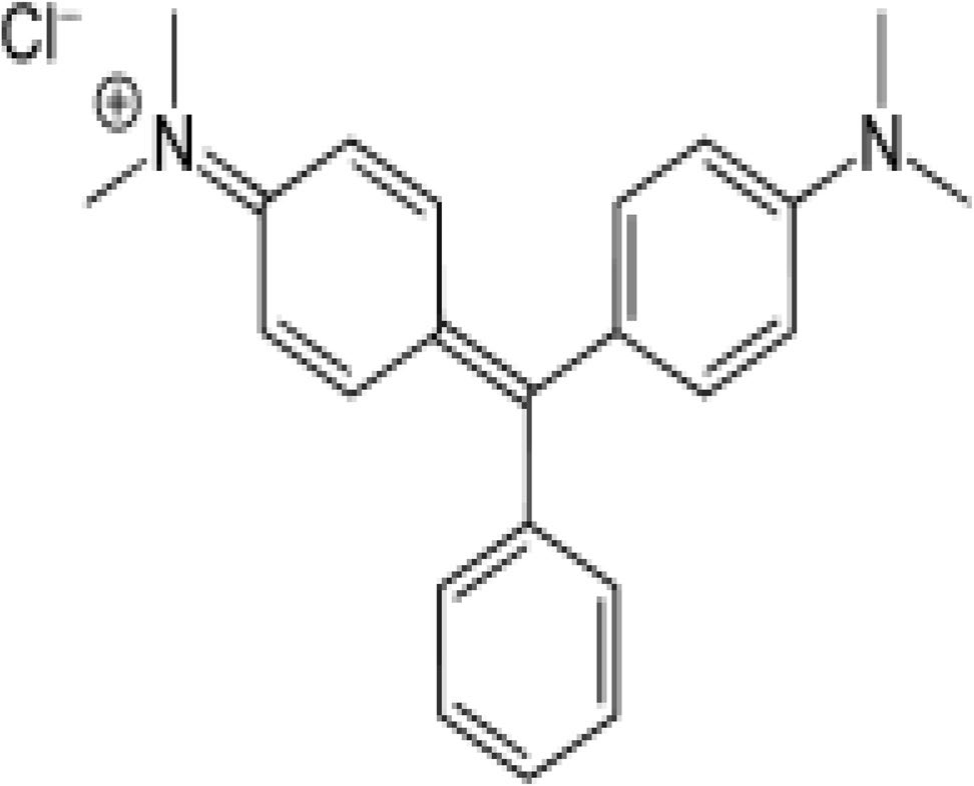
Malachite Green dye structure.

Analytical grade reagents were used all through the study. Stock solution of 1000 mg/L MG dye (Fig. 1) solution was prepared by dissolving 1 g MG salt in 1000 mL distilled water. A lower working concentration was prepared (100–600 mg/L) by serial dilution.

#### Biosorption operational parameters

Various Operational parameters relevant to this study were carried out following reported method^3,4,35^. The effect of pH was determined by varying the pH values between 3 and 10 via dropwise addition of 1 M HCl or NaOH where applicable. The effect of initial MG concentration was investigated by the introduction of 1 g AFMC into different concentrations of MG dye (100–600 mg/L). Variation of time as done to investigate the effect of contact time from 10 to 120 min. All through the study, the adsorbate-adsorbent system was agitated on the Orbital shaker to increase effective collision in the system. Measurement of residual concentration at maximum wavelength of 617 nm was done using double beam Libra Biochrom 5505 v1.0.4 PCB 1500 coupled with water Peltier system UV–Vis spectrophotometer.

### Theory

#### Biosorption isotherm and kinetic modeling and statistical error validity

Equilibrium biosorption data obtained from the study of were analyzed using six of two-parameter models (Freundlich^36^, Langmuir^16^, Temkin^37^, Dubinni-Raduskevich^38^, Halsey^39^ and Jovanovic^40^). Similarly, both kinetics and mechanism models were fitted to Pseudo first-order^41^, Pseudo-second-order^42^, Elovich^43^, Fractional power^44^, Intraparticle^45^ and liquid film^46^ diffusion models. Estimation of the quantity adsorbed and percentage removal efficiency was done using Eqs. (4) and (5)^47–49^

4 $$Q_{e} = \frac{{\left( {c_{o} - c_{e} } \right)v}}{w}$$

5 $$\% RE = \frac{{c_{o} - c_{e} }}{{c_{o} }} \times 100$$

Presented in Tables 1 and 2 are the descriptions of both isotherm, kinetics, and mechanism models used in this study.

**Table 1:** Adsorption Isotherm Models (Non-linear and linear models with the description of parameters)^46–50^.

Types of adsorption models	Non-linear expression	Linear expression	Parameters nomenclature and description
Langmuir	${Q}_{e}=\frac{{Q}_{max}{K}_{L}{C}_{e}}{1+{K}_{L}{C}_{e}}$	$\frac{1}{{{\varvec{Q}}}_{{\varvec{e}}}}=\frac{1}{{{\varvec{Q}}}_{{\varvec{m}}{\varvec{a}}{\varvec{x}}}}+\frac{1}{{{\varvec{Q}}}_{{\varvec{m}}{\varvec{a}}{\varvec{x}}}{{\varvec{K}}}_{{\varvec{L}}}{\varvec{C}}{\varvec{e}}}$ (6) ${\mathrm{R}}_{\mathrm{L}}=\frac{1}{{{1+K}_{L}C}_{o}}$ (7)	K_L_ is the Langmuir isotherm constant (L/mg) related to the binding energy of adsorption.${Q}_{max}$ is the maximum monolayer coverage capacity (mg/g), R_L_ dimensionless separation factor indicating the nature and favourability of adsorption process. From slope and intercept of linear plot of Ce/Qe vs 1/Ce, K_L_ and Q_max_ were determined
Freundlich	${Q}_{e }={K}_{F}{C}_{e}$	$log{Q}_{e}=log{K}_{F}+\frac{1}{{n}_{F}} log{C}_{e}$(8)	*C*_*e*_ equilibrium concentration of the MG dye adsorbate (mgL^-1^); Q_e_ amount of MG dye adsorbed at equilibrium per unit weight of AFMC (mg g^-1^); *K*_F_ Freundlich indicator of adsorption capacity,1/n_F_ Intensity of the adsorption indicating the surface heterogeneity and favourability of the adsorption process 1/n_F_ and *K*_F_ were determined from slope and intercept of linear plot of log Qe vs log Ce
Temkin	${Q}_{e}=\frac{RT}{{b}_{T}} ln\left({A}_{T}{C}_{e}\right)$	${Q}_{e}=\frac{RT}{{b}_{T}}ln{A}_{T}+\frac{RT}{{b}_{T}}ln{C}_{e}$(9)	***b***_***T***_ is the Temkin isotherm constant related to the heat of adsorption and A_*T*_ is the Temkin isotherm equilibrium binding constant (L/g) R = universal gas constant (8.314 J/mol/K) T = absolute Temperature in Kelvin B = RT/b_T_ = Constant related to heat of sorption (J/mol) obtained either from intercept or slope
DKR	${Q}_{e}={Q}_{DKR}{exp}^{{-A}_{D-R}}{\varepsilon }^{2}$	$\ln{q}_{e}=\ln{Q}_{DKR}-A{{}_{DKR}\varepsilon }^{2}$(10) $\varepsilon =RT\ln\left[1+\frac{1}{{C}_{e}}\right]$ (11) $E=-\left[\frac{1}{\sqrt{{2A}_{D-R}}}\right]$ (12)	Q_DKR_ is the theoretical adsorption isotherm saturation capacity (mg/g) obtained from intercept. A_DkR_ is the D–R isotherm constant (mol^2^/kJ^2^) related to free sorption energy obtained from the slope. Ɛ is Polanyi potential determined by the expression = RT ln(1 + 1/C_e_). E is the mean adsorption free energy helpful in determining the adsorption nature (physisorption or chemisorption of the adsorption process). Q_D-R_ and A_D-R_ were determined from intercept and slope of linear plot of ln q_e_ vs Ɛ^2^
Halsey	${Q}_{e}=exp\left[\frac{{lnK}_{H}-{lnC}_{e}}{{n}_{H}}\right]$	$log{Q}_{e}=\left[\left(\frac{1}{{n}_{H}}\right) ln{K}_{H}\right]-\left(\frac{1}{{n}_{H}}\right) ln{C}_{e}$(13)	K_H_ is Halsey isotherm constant; n_H_ is the Halsey isotherm exponent. Both were determined from linear plot of logQe vs ln Ce
Jovanovic	${Q}_{e}={Q}_{J}\left[{1-exp}^{{(K}_{J}{C}_{e})}\right]$	$ln{Q}_{e}={lnQ}_{max}-{K}_{J}{C}_{e}$(14)	K_J_ is Jovanovic isotherm constant (L/g) determined from the slope of plot of ln *q*_*e*_ against C_e_

**Table 2:** Kinetics and mechanism modeling of adsorption^40–51^.

Kinetic and mechanism models	Linear expression	Parameters nomenclature and description
Pseudo first order (PFO)	$log\left({q}_{e}-{q}_{t}\right)={logq}_{e}-\frac{{K}_{1}t}{2.303}$ (15) ${h}_{1}={k}_{1}{q}_{e}$ (16)	q_e_ is the quantity of adsorbate at equilibrium per unit weight of the adsorbent (mg/g), q_t_ is the amount of adsorbed at any time (mg/g) and k_1_ is the pseudo first-order rate constant (min^−1^) and h_1_ initial pseudo first-order rate constant (mg/g/min). q_e_ and k_1_ were determined respectively from intercept and slope of the linear plot of loq_e_-q_t_ vs t
Pseudo second-order (PSO)	$\frac{t}{{q}_{t}}=\frac{1}{{k}_{2}{q}_{e}^{2}}+\frac{t}{{q}_{e}}$(17) ${h}_{2}={k}_{2}{q}_{e}^{2}$ (18)	k_2_ is the pseudo second-order rate constant (min^−1^) h_2_ is initial pseudo second-order adsorption rate constant (mg/g/min). q_e_ and k_2_ were determined respectively from slope and intercept of the linear plot of t/q_t_ vs t
Elovich	${q}_{t}=\frac{1}{\beta }\ln\left(\alpha \beta \right)+\frac{1}{\beta }\ln\left(t\right)$(19)	q_t_ is the amount of adsorbate per unit mass of adsorbent at time (t), and α and β are the constants slope and intercept of the determined from the linear plot of q_t_ versus ln(t). ∝ is the initial adsorption rate (mg/g-min); β is the desorption constant (g/mg) during any one experiment. The slope is $1/\beta$ while the intercept is $1/\beta\ln(\alpha \beta )$
Fractional power (power function)	$\log\hspace{0.33em}({q}_{t})=\mathit{log}(k)+v\mathit{log}(t)$(20)	q_t_ is the amount of adsorbate per unit mass of adsorbent, k is a constant, t is time, and v is a positive constant (< 1). The parameters v and k are obtainable from slope and intercept of a linear plot of log (q_t_) versus log (t)
Intraparticle diffusion (IPD)	${q}_{t}={k}_{id}{t}^{0.5}+C$(21)	k_id_ is the intraparticle diffusion rate constant (mg.g^−1^min^0.5^) and C is the thickness of the adsorbent determined from slope and intercept of linear plot of q_t_ vs t^0.5^
Liquid film diffusion (LFD)	$\mathit{ln}\left(1-F\right)=-{K}_{LFD}t$+C (22) $F=\frac{\left[{q}_{t}^{n}\right]}{\left[{q}_{e}^{n}\right]}$ (23)	F is fractional attainment to equilibrium and K_LFD_ is the rate coefficient for particle-diffusion controlled process corresponding to the particle size of the adsorbent. -K_LFD_ was determined from the linear plot of ln(1 − F) vs t

#### Adsorption statistical error function (ASRF) models

In most cases, determination of best fitting relationship and finalizing the best isotherm and kinetics model have always been through the use of linear correlation coefficient (*R*^2^) values. However. Owing to inherent bias from this transformation, the following four rigorous statistical error function models were used: Sum of square error (SSE)^50^; Hybrid fractional error function (HYBRID)^39^; Nonlinear chi-square test (χ^2^)^51^; Marquardt’s Percent Standard Deviation (MPSD)^52^, Presented in Table 2 are the equation of the Adsorption Statistical Error Function (ASRF) Models from Eqs. (24)–(27).

24 $${\text{Sum }}\;{\text{of }}\;{\text{Square }}\;{\text{Error}}\;{\text{SSE}} = \mathop \sum \limits_{i = 1}^{n} \left( {q_{e,cal} - q_{e,\exp } } \right)^{2}$$

25 $${\text{Non - linear - chi - square}}\;{\text{ test}}\;\chi^{2} = \mathop \sum \limits_{i = 1}^{n} \frac{{\left( {q_{e,cal} - q_{e,\exp } } \right)^{2} }}{{q_{e,cal} }}$$

26 $${\text{Hybrid}}\;{\text{ fractional }}\;{\text{error }}\;{\text{functions}}\;HYBRID = \sum\limits_{i = 1}^{n} {\left[ {\frac{{\left( {q_{e,\exp } - q_{e,cal} } \right)^{2} }}{{q_{e,\exp } }}} \right]}_{i}$$

27 $${\text{Marquardt's}}\;{\text{ Percent }}\;{\text{Standard }}\;{\text{Deviation}}\; \, \left( {MPSD} \right)MPSD = \mathop \sum \limits_{i = 1}^{n} \left[ {\frac{{\left( {q_{e,\exp } - q_{e,cal} } \right)}}{{q_{e,\exp } }}} \right]^{2}$$

Both isotherm and kinetics data were tested with the statistical error validity models.

## Results and discussion

### Physicochemical characterization

Figure 1 shows the structure of Malachite green as a cationic dye. Presented in Table S1 of the supplementary document is the physicochemical characteristics of Malachite Green (MG) indicating that it is a cationic dye having vast application. The unique physicochemical properties of AFMC were determined and summarized in Table S2. The pH determined was 6.75, surface area (1329 m^2^/g), 12% moisture content, 0.386 g/cm^3^ bulk density, and approximated particle size 300 μm < Ф < 250 μm. It has been reported that for applicability, activated carbon in the range of pH 6 to 8 is acceptable. The pH of AFMC determined as 6.75 is suitable for activated carbon (AC). The amount of water bound to activated carbon is determined via the moisture content. Lower moisture content is desirable for active activated carbon because of the competition of the water vapor with the pores of AC. The moisture content of AFMC lower than commercial activated carbon (CAC)^53^ is suitable. The filterability of activated carbon is determined from the bulk density.

### Effect of pH

Shown in Fig. S1 is the effect of pH on biosorption of MG cationic dye onto AFMC. Ionic mobility and degree of ionization as well as the surface chemistry was influence by this operational parameter. Protonation, as well as ionic competition between H^+^ and MG^+^ zwitterion in aqueous solution for available sites, was observed between pH 2–5 at the acidic region. Higher quantity adsorbed and removal efficiency observed between pH 6 and 8 was due to deprotonation, low competition, and a higher aggregate of MG^+^. 7.425 mg/g quantity of MG was adsorbed at 100 ppm as observed at pH 6. Beyond pH 6, no further increase was observed, therefore pH 6 was chosen as an optimum pH similar to the finding of Alqadami et al*.* where MOF was used for adsorption of both Malachite green and methylene blue and optimum pH for highest adsorption capacity of MG was at 6.8^28^.

### Effect of initial concentration

Figure S2 of the supplementary document shows the result of the effect on initial concentration on effective removal of EDC cationic MG dye using AFMC. Lower transport of the MG dye at lower concentrations led to lower adsorption due to low driving force. However, the percentage of the percentage removal efficiency increased with increase in concentration. The concentration gradient developed was due to bombardment of the MG^+^ surrounding the active sites. It is obvious from Fig. S2 that rapid adsorption was observed at low concentration as a result of an increase in the active sites as compared to MG molecules in the bulk. Thereafter, diffusion, convection, and migration of MG molecules as a result of mass transport from the bulk lead to an increase in removal efficiency until a saturated point was reached. All the active sites were filled up at equilibrium and thereafter, no significant percentage removal efficiency observed. Similar finding was observed by Khan et al. and in the literature^29,46,47^.

### Biosorption Isotherm modeling and statistical validity

**Figure 2:**
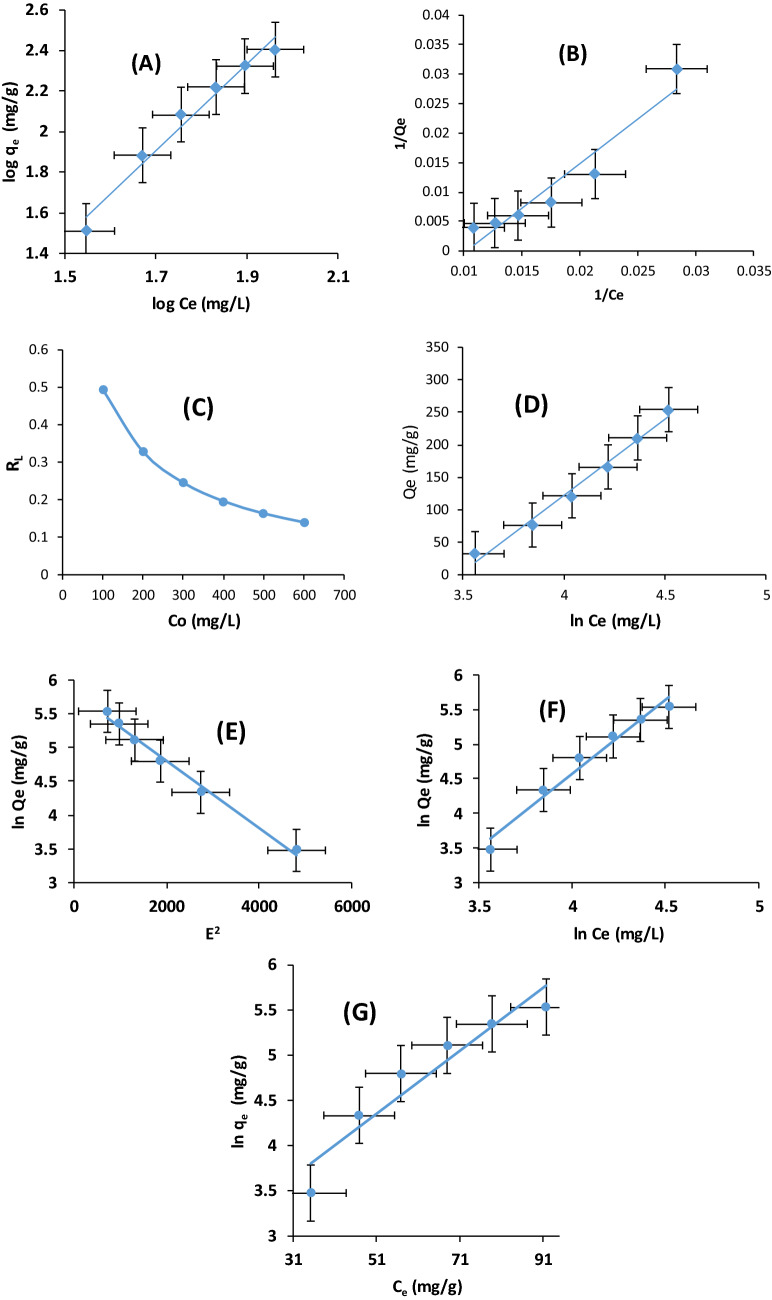
(**A**–**G**) Plots of: (**A**) Freundlich isotherm model, (**B**) Langmuir isotherm model, (**C**) Separation Factor on sorption of MG onto AFMC, (**D**) Temkin isotherm model, (**E**) D–R isotherm model, (**F**) Halsey isotherm model, (**G**) Jovanovic isotherm model for sorption of MG dye onto AFMC.

Understanding of the binding interaction between AFMC and MG dye solution is enhanced by the study of the isotherm models. Equilibrium data were fitted to six isotherm models namely; Freundlich (Fig. 2A), Langmuir (Fig. 2B), Temkin (Fig. 2D), Dubinin-Raduskevich (Fig. 2E), Halsey (Fig. 2F), and Jovanovic (Fig. 2G). Portrayed in Fig. 2A–G are the isotherm models’ linear plots. Better fit with R^2^ > 0.97 were observed in Table 3a for Freundlich, Temkin, Dubinin–Raduskevich (D–R), Halsey. Equilibrium data did not fit well to Langmuir and Jovanovic considering their R^2^ value less than 0.92 (Table 3). Both Freundlich and Halsey isotherm models describe the adsorption characteristic for the heterogeneous surface. The characteristics parameters of Freundlich isotherm models are K_F_ (adsorption capacity) and 1/n_F_ and n_F_ (adsorption intensity) obtained from the linear plot of log Qe against log Ce. The function of the strength of adsorption of MG onto AFMC is determined from the parameter 1/n_F._ The value of 1/n_F_ (2.1372) being above unity is an indication of a cooperative adsorption^54,55^. The favourability of the adsorption process of MG onto AFMC could be affirmed from the Langmuir dimensionless and separation factor (R_L_). The R_L_ value indicates the adsorption nature to either unfavorable or unfavorable. It is unfavourable if R_L_ > 1, linear if R_L_ = 1, favourable if 0 < R_L_ < 1 and irreversible if R_L_ = 0. The value of R_L_ ranges between 0.00377 and 0.0744 and being less than one indicated favorable adsorption. There are many studies carried out on the adsorption of MG onto different adsorbents. Comparison of the Qmax monolayer capacities of adsorption of MG onto various adsorbents was presented in Table 4. Qmax of AFMC surpassed all those adsorbents compared indicating that AFMC is a better adsorbent for MG adsorption.

**Table 3:** Isotherm models’ parameters and for adsorption of malachite green onto AFMC.

Type of isotherm	Model parameters	Evaluated value
Freundlich	Model parameters	Evaluated value
	k_f_	2.1373
	1/n_f_	2.1373
	n_f_	0.4679
	R^2^	0.9726
Langmuir	Parameters	Values
	Q_max_ (mg/g)	64.5161
	K_L_ (L/mg)	0.01023
	R_L_	0.140–0.494
	R^2^	0.9149
Temkin	Parameters	Values
	b_T_ (J/mol)	10.555
	β (L/g)	234.72
	A_T_ (L/g)	0.03078
	R^2^	0.9846
D–R	Parameters	Values
	Q_d_	330.135
	A_DKR_	5 × 10^–4^
	E (J/mol)	3.163 × 10^1^
	R^2^	0.989
Halsey	Parameters	Values
	1/n_H_	− 2.1373
	n_H_	− 0.4679
	K_H_	6.4286
	R^2^	0.9726
Jovanovic	Parameters	values
	Q_max_	13.1615
	K_j_	0.0348
	R^2^	0.9082

**Table 4:** Comparison of the Qmax monolayer capacity of MG adsorption with other adsorbents.

S/N	Adsorbents	Maximum monolayer capacity	References
1	Chitosan–zinc oxide composite	11	^10^
2	SSB/Fe–Cu	63.5	^15^
3	*Carica papaya* wood	52.63	^27^
4	Native shells of Peltophorum pterocarpum	40	^40^
5	NaOH activated shells of Peltophorum pterocarpum	62.5	^40^
6	Carbonized pomegranate peel	31.45	^58^
7	Fe_2_O_3_ by ODH	15.72	^65^
8	Fe_2_O_3_ by PEG	16	^65^
9	AFMC	64.52	This study

The Dubinin–Kaganer–Raduskevich is generally applied to determine the mechanism of the MG-dye and AFMC system with a Gaussian energy distribution onto a heterogeneous surface. The R^2^ > 0.98 is an indication of a better description of equilibrium data by The DKR mean energy (E) value being less than 8 kJ indicated that the mechanism is physisorption. Studies from Bello et al*.* on scavenging of MG onto *Citrus grandis* peels further supported these findings^38,56^.

### Statistical error validity on isotherm model

Studies have shown that the determination of the best isotherm model does not only depend on the R^2^ value. Statistical validity model has been introduced to further justify the suitability of the best isotherm model to describe the adsorption process^49,59^. Table 5 has shown the values of the experimental and calculated quantity adsorbed, Qe, exp and Qe, cal, respectively. The four most used statistical validity models in adsorption studies explored are SSE, HYBRID, X^2^, and MPSD. Adsorption Statistical Error Function (ASRF) has always been the most reliable validity parameter in justifying the best isotherm model for the equilibrium studies. To determine the isotherm best model, coupled with the higher R^2^ value, there must be closeness between the data of the Qe, cal, and Qe, exp alongside a low value of the ASRF^60,61^. Considering Table 5, Freundlich, Temkin, and Halsey isotherm models fit well into these conditions for fitness. From Table 4, the R^2^ values (0.9726 for Freundlich, 0.9726 for Halsey and 0.9846 for Temkin) are closer to unity with consistent agreement between Qe, exp and Qe, cal (254.13 mg/g and 293.43 mg/g for Freundlich; 254.13 mg/g and 243.75 mg/g for Temkin; 254.13 mg/g and 293.51 mg/g for Hasley).

**Table 5:** Adsorption statistical error function (ASRF) data on adsorption isotherm models.

ASRF models	Freundlich	Langmuir	Temkin	D–R	Halsey	Jovanovic
q_e_,_exp_ (mg/g)	254.13	254.13	254.13	254.13	254.13	254.13
q_e_, _cal_ (mg/g)	293.43	36.3703	243.75	29.81	293.51	320.7
R^2^	0.9726	0.9149	0.9846	0.989	0.9726	0.9082
SSE	1544.49	47,419.29	107.7444	50,319.46	1550.784	4431.565
HYBRID	6.077559	186.5946	0.423974	198.0068	6.102327	17.43818
X^2^	5.263572	1303.791	0.442028	1688.006	5.283583	13.81841
MPSD	0.023915	0.734249	0.001668	0.779155	0.024013	0.068619

### Effect of contact time at various initial concentrations

Importance relevant parameter that controls the transfer and build-up of charges from the bulk to the pore active site in all transfer media is the contact time. Effect of contact time was studied from 10 to 120 min at six different initial concentrations from 100 to 600 mg/L as depicted in Fig. S3 of the supplementary document. Based on the results, rapid adsorption was observed in the first 30 min dues increase attractive forces between the active sites and MG molecules as a result of van der Waals forces and electrostatic attractions. Between 60 and 90 min not significant increase in adsorption capacity and removal, efficiency was observed due to attainment of saturation and equilibrium. A fast diffusion onto the external surface of AFMC was followed by fast pore diffusion into the intraparticle matrix as a result of the participation of the functional groups until equilibrium was attained where 93.09% removal efficiency was achieved. The reaction was allowed to proceed till 90 min beyond which to increase was observed as depicted in Fig. 5. This finding is supported by the report of Hamdaoui et al.^57^ as well as Figen and Bayrak^58^.

### Kinetics and mechanism model of MG sequestration

**Figure 3:**
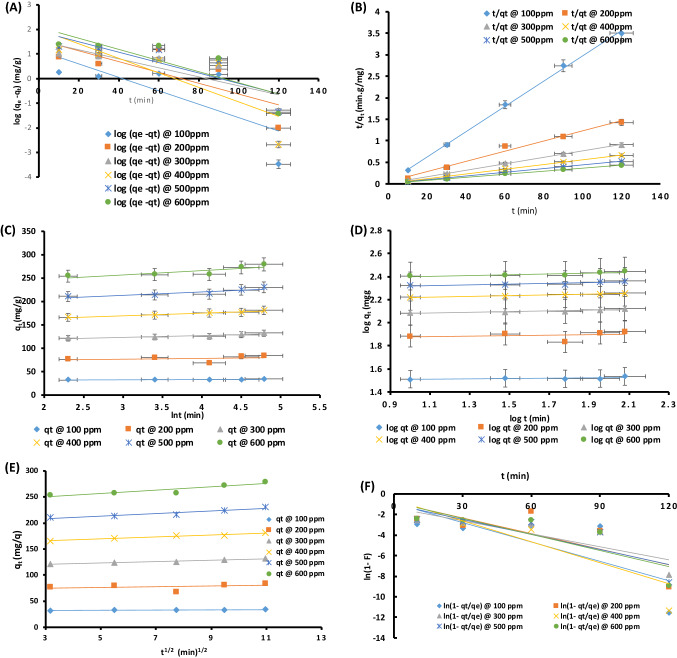

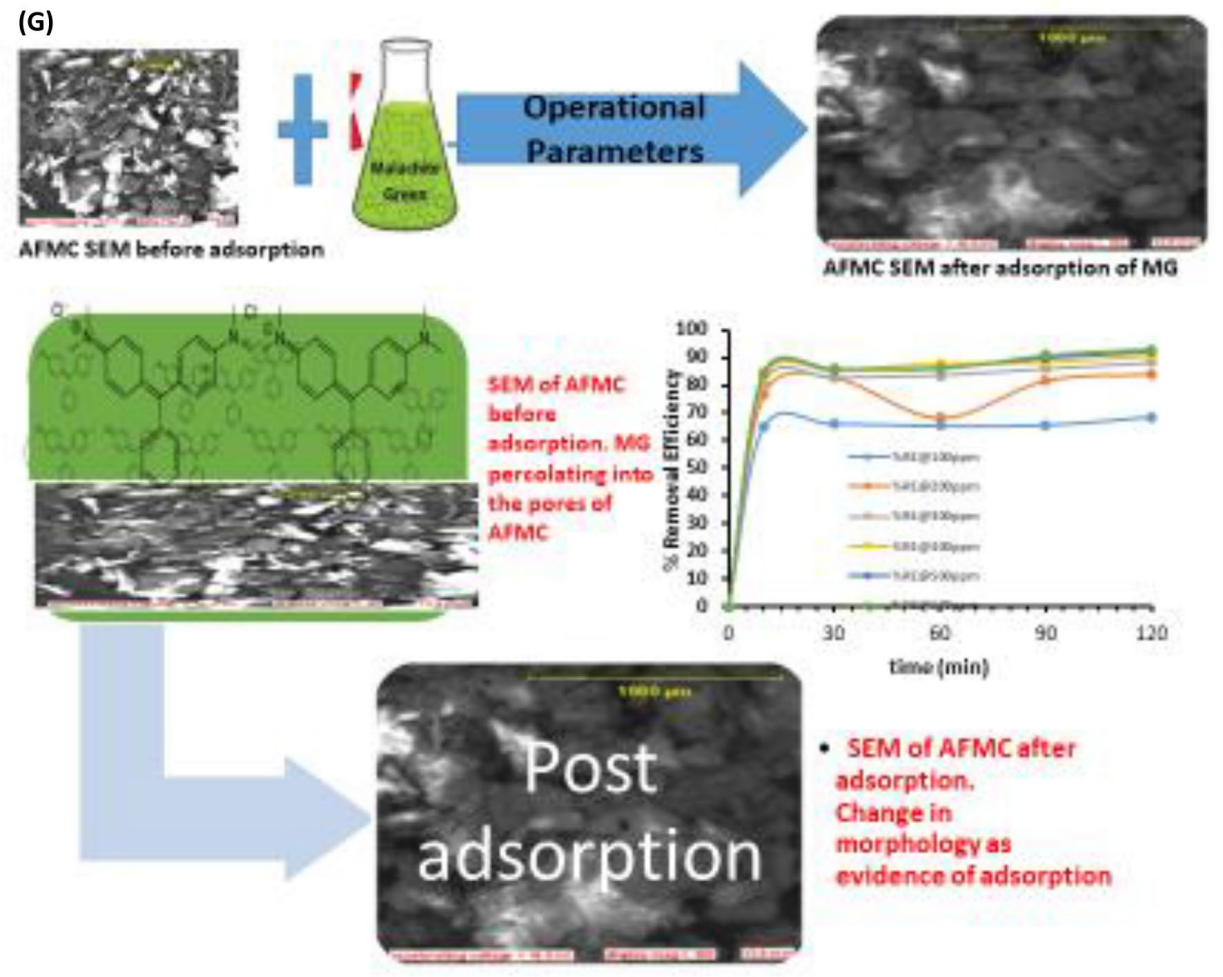
(**A**) Pseudo first-order kinetics model (Conditions; pH 6, AFMC dose = 100 mg, contact time:90 min; concentrations: 100–600 mg/L). (**B**) Pseudo second-order kinetics model (Conditions; pH 6, AFMC dose = 100 mg, contact time:90 min; concentrations: 100–600 mg/L). (**C**) Elovich kinetics model (Conditions; pH 6, AFMC dose = 100 mg, contact time: 90 min; concentrations: 100–600 mg/L). (**D**) Fractional power kinetics model (Conditions; pH 6, AFMC dose = 100 mg, contact time:90 min; concentrations: 100 – 600 mg/L). (**E**) Intraparticle diffusion model (Conditions; pH 6, AFMC dose = 100 mg, contact time: 90 min; concentrations: 100–600 mg/L). (**F**) Liquid film diffusion mechanism model (Conditions; pH 6, AFMC dose = 100 mg, contact time: 90 min; concentrations: 100–600 mg/L). (**G**) Schematic diagram of the adsorption of MG dye onto AFMC (AFMC is depicted by its AFMC SEM morphology).

The rate of binding of MG onto AFMC was determined by the adsorption kinetics which also helps in gaining insight into the mechanism of the sorption process. Across various concentrations from 100 to 600 mg/L, the kinetic data were fitted to the following kinetics and mechanism models: Pseudo first-order (PFO)(Fig. 3A), Pseudo second-order (PSO) (Fig. 3B), Elovich (Fig. 3C), Fractional power (power function) (Fig. 3D); Intraparticle Diffusion (Fig. 3E) and Liquid film diffusion (Fig. 3F). Based on the evaluated data presented in Table 6, correlation coefficient *R*^2^ of pseudo-second-order (> 0.99) is highest among all the kinetics models explored. The R^2^ value is consistently higher and increases as the concentration increases. The h_2_ initial pseudo-second-order adsorption rate constant increases from 23.92 to 105.26 mg/g/min suggesting a rapid kinetic process*.* The error bars on the kinetic plots from Fig. 3A–D showed that the kinetic models were validated using statistical error functions. The consistency of the calculated adsorption capacity (Q_e_, cal) with the experimental adsorption capacity (Q_e_, exp) coupled with lower values of the statistical error function validity data as observed in SSE, HYBRID, X^2^_,_ and MSPD further supported the PSO as the best kinetic model in this study. This result is supported by the investigation carried out by researchers^59,60^.

**Table 6:** Parameters of kinetics models with associated Statistical Validity Data.

Kinetics model parameters			Various concentrations			
	100 mg/L	200 mg/L	300 mg/L	400 mg/L	500 mg/L	600 mg/L
**Pseudo first-order**						
q_e_,exp (mg/g)	34.243	84	132.3	181.02	230.3	279.3
q_e_, cal (mg/g)	13.9284	38.256	34.546	101.555	83.483	124.48
k_1_(min^−1^)	0.0621	0.0506	0.0414	0.05	0.0407	0.5269
h_1_ (mg/g/min)	0.864954	1.935754	1.430204	5.07775	3.397758	65.58851
R^2^	0.5443	0.5827	0.7156	0.6609	0.6989	0.689
SSE	412.683	2092.514	9555.845	6314.687	21,555.23	23,969.23
HYBRID	12.0516	24.91088	72.22861	34.88392	93.59631	85.81895
X^2^	29.62889	54.69767	276.6122	62.17997	258.199	192.5549
MPSD	0.351943	0.296558	0.545946	0.192708	0.40641	0.307264
**Pseudo Second-order**						
q_e_, exp (mg/g)	34.243	84	132.3	181.02	230.3	279.3
q_e_, cal (mg/g)	34.1296	84.746	133.333	181.8182	232.5581	285.7143
k_2_(g/mg/min)	0.02054	0.00276	0.003583	0.00318	0.00174	0.00123
h_2_ (mg/g/min)	23.9234	19.8412	63.694	105.263	94.339	101.0101
R^2^	0.9986	0.9833	0.9992	0.9996	0.9989	0.9983
SSE	0.01286	0.556516	1.067089	0.637123	5.099016	41.14324
HYBRID	0.000376	0.006625	0.008066	0.00352	0.022141	0.147308
X2	0.000377	0.006567	0.008003	0.003504	0.021926	0.144001
MPSD	1.1 × 10^–5^	7.89 × 10^–5^	6.1 × 10^–5^	1.94 × 10^–5^	9.61 × 10^–5^	5.27 × 10^–4^
**Elovich**						
q_e_,exp (mg/g)	34.243	84	132.3	181.02	230.3	279.3
q_e_, cal (mg/g)	33.468	79.753	130.269	179.519	226.119	273.279
α(g min^2^/mg)	8.95 × 10^27^	10.3 × 10^17^	9.26 × 10^12^	2.42 × 10^12^	1.93 × 10^12^	0.494 × 10^12^
β(g.min/mg)	2.0881	0.5732	0.2555	0.1758	0.1375	0.1079
R^2^	0.4294	0.0791	0.8596	0.9643	0.7837	0.699
SSE	0.600625	18.03701	4.124961	2.253001	17.48076	36.25244
HYBRID	0.01754	0.214726	0.031179	0.012446	0.075904	0.129797
X2	0.017946	0.226161	0.031665	0.01255	0.077308	0.132657
MPSD	0.000512	0.002556	0.000236	6.88E−05	0.00033	0.000465
**Fractional power**						
q_e_,exp (mg/g)	34.243	84	132.3	181.02	230.3	279.3
q_e_, cal (mg/g)	33.461	79.463	130.303	179.558	226.059	273.189
v(min^-1^)	0.0144	0.021	0.031	0.0329	0.0331	0.0349
k_3_ (mg/g)	31.232	71.8621	112.331	153.391	192.93	231.153
k_3_v(mg/g/min)	0.449741	1.509104	3.482261	5.046564	6.385983	8.06724
R^2^	0.4325	0.0651	0.8676	0.9686	0.7911	0.7043
SSE	1118.675	6311.031	16,970.79	32,229.26	51,087.71	74,613.16
HYBRID	33.43221	79.42101	130.241	179.4922	225.9928	273.1192
X2	77,685.77	300,525.3	547,445	979,612.8	1,543,435	2,137,913
MPSD	0.999139	0.999472	0.999524	0.999634	0.999707	0.999745

Presented in Fig. 3D is the Fractional power plot for adsorption of MG onto AFMC. Considering Table 6, the parameters v and k being positive, greater than unity, and increase with the increase in concentration suggested a rapid kinetic process. The close agreement between Q_e_ exp and Q_e_, cal are indications of the best fitting of the kinetic data to the fractional power model. At low concentration, the R^2^ values were far away from unity, however, better regression coefficients were obtained with higher concentration indicating the applicability of the adsorbent, AFMC, to the removal of pollutant at higher concentrations of MG dye. The choice of the best fit kinetic model was adjudged not only with correlation coefficient but also with the statistical error validity functions. It has been established that the model with a higher R^2^ value, nearness/closeness between Q_e_,exp and Q_e_, cal and lower data of statistical error function, would be chosen as the best descriptive model^61–64^. Pseudo second-order fit perfectly well into this condition and thus the best kinetic model to describe the sequestration of MG dye onto AFMC. Supporting this claim is the finding of Dehbi et al.^65^.

Figure 3E and F show the linear plots of Intraparticle Diffusion (IPD) and Liquid film diffusion (LFD) models. The evaluated parameters are presented in Table 7. Both the rate-controlling step and the diffusion mechanism were explored using IPD because its R^2^ values were consistently higher than that of the LFD. IPD would be the only rate-determining step if its plot begins from the origin. Contrary to this, the plot of q_t_ against t^1/2^ did not begin from the origin hence IPD is not the only rate-determining step. However, the value of the thickness C of the adsorbent calculated from the IPD model being greater than zero across all concentrations indicated that the thickness of the boundary layer participated in the adsorption process. It is suggested that since boundary layer, C > 0 from the evaluated parameters in Table 7, another diffusion model may be involved in determining the rate-controlling step^65^.

**Table 7:** Mechanism model parameters of adsorption of MG onto AFMC.

Mechanism models	Mechanism model parameters of adsorption of MG onto AFMC					
	Various Concentrations					
Parameters	100 mg/L	200 mg/L	300 mg/L	400 mg/L	500 mg/L	600 mg/L
**Intraparticle diffusion**						
k_ip_(mg/g/min^0.5^)	0.1671	0.7151	1.3105	1.8328	2.481	3.209
C	31.772	72.793	116.82	160.5	200.79	240.6
R^2^	0.513	0.1304	0.945	0.9823	0.8944	0.822
**Liquid film diffusion**						
K	0.0628	0.0506	0.0421	0.0679	0.0487	0.0521
C	0.899	0.814	1.343	0.578	1.015	0.808
R^2^	0.544	0.583	0.716	0.661	0.699	0.689

As reported by Dada et al.^4,62^ and Boparai et al.^61^, three definite steps involved in adsorption are: intraparticle or pore diffusion, where adsorbate molecules percolates into the interior of adsorbent particles; Liquid film or surface diffusion where the adsorbate is transported from the bulk solution to the external surface of adsorbent, and adsorption on the interior sites of the adsorbent. From this study, Pseudo second order (PSO) model best described the kinetic data as a result of a rapid adsorption process which is being supported by best R^2^ values and low statistical validity models. More so, the mechanism is pore diffusion dependent. Depicted in Fig. 3G is the scheme of mechanism of adsorption of malachite green onto AFMC. Adsorption is always a surface phenomenon. This scheme shows the summary of the adsorption of MG onto AFMC. Change in morphology was noticed after adsorption showing the evidence of the percolation of the MG dye into the pores and matrix of AFMC.

### Surface morphology and surface chemistry post adsorption characterization

**Figure 4:**
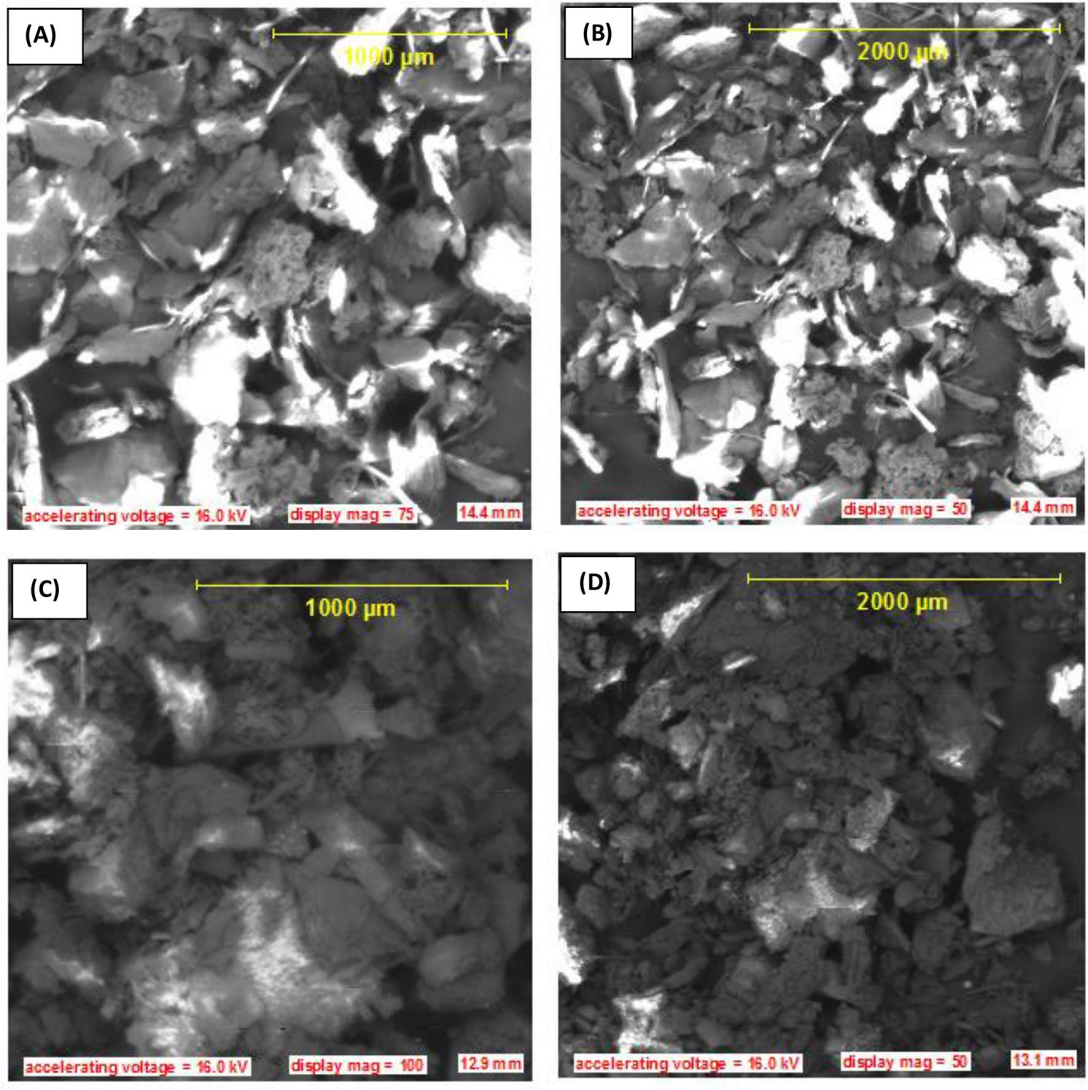
(**A**, **B**) SEM morphology of AFMC before adsorption at 1000 μm and 2000 μm magnifications. (**C**, **D**): SEM morphology of AFMC after adsorption at 1000 μm and 2000 μm magnifications.

Evidence of the adsorption process was justified by morphological characterization of AFMC before and after adsorption onto MG using scanning electron microscopy (SEM). More so, surface chemistry was investigated by functional group determination using Fourier Transform Infrared (FTIR) spectroscopy. Before adsorption, dry and particle-like-crake nature with the presence of pores is evident all through the micrographs at different magnifications as portrayed in Fig. 4A,B. However, after adsorption as depicted in Fig. 4C,D, there was the disappearance of crakes, impregnated pores with MG dye solution, and robustness of AFMC adsorbent are morphology evidence of the adsorption process.

**Figures 5:**
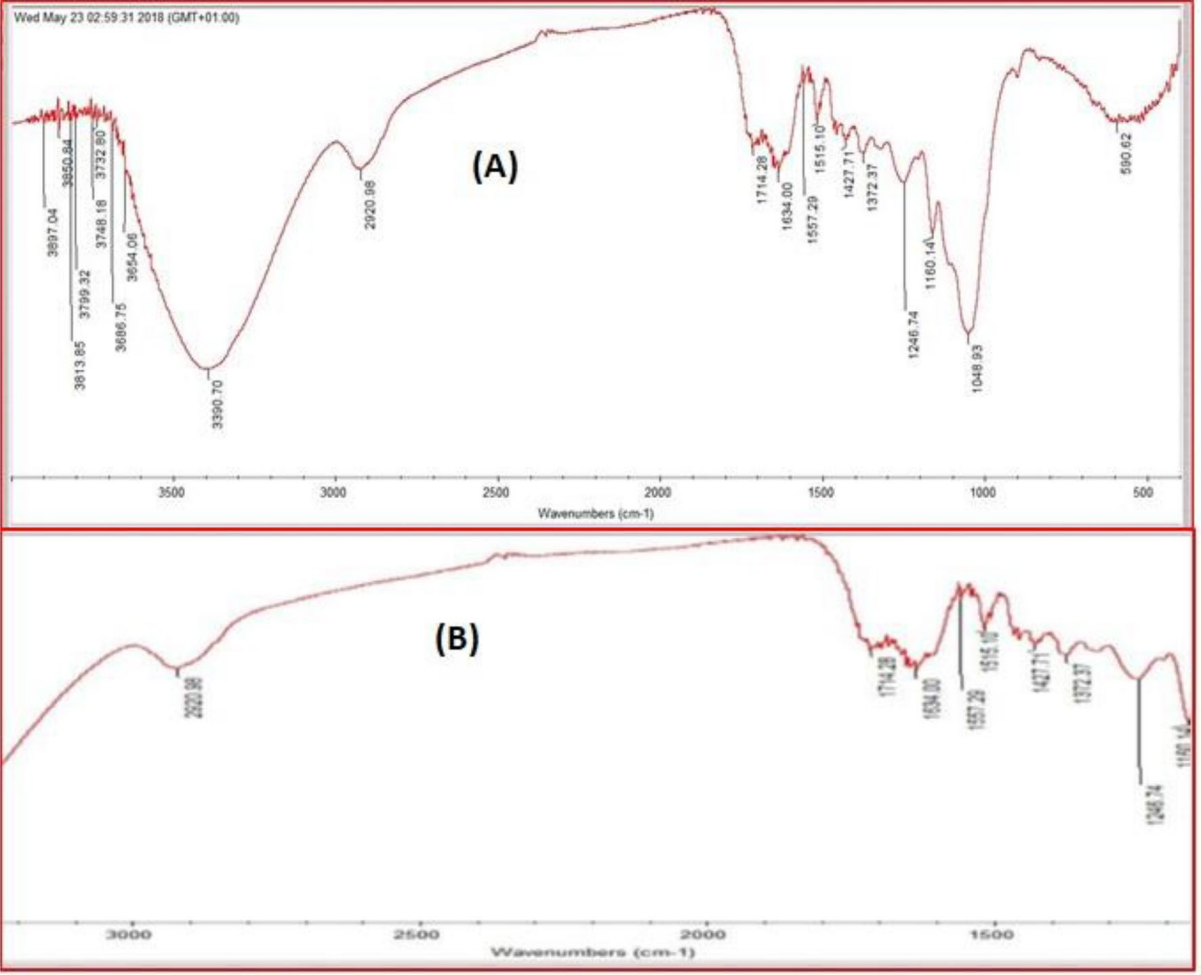
(**A**) FTIR spectrum before adsorption of MG dye, (**B**) FTIR spectrum after adsorption of MG dye.

Depicted in Fig. 5A,B are the FTIR spectra of AFMC before and after adsorption. The surface chemistry of AFMC before and after adsorption was investigated using FTIR. Broadband at 3390.70 cm^−1^ is attributed to O–H stretching of the hydrogen bonding which disappeared after adsorption as evidence of its participation in the adsorption process^66^. Aliphatic C–H stretching band at 2920.98 cm^−1^ was also found to decrease after adsorption. Carbonyl group, –C=O stretching vibration attributed to the lignin aromatic groups was assigned to 1714.28 cm^−1^ and 1667.29 cm^−1^. Ascribed to –C=C– bending of the Aromatic ring are the signals observed between 1515.10 and 1427.71 cm^−1^ while –CH_3_ bands as a result of deformation are observed at 1372.37 cm^−1^. Several bands between 1200 and 800 cm^−1^ are ascribed to characteristic carbohydrate bands while at 1049.93 cm^−1^, C–O vibrational band assigned to cellulose is observed at 1049.93 cm^−1^. The shift in bands and disappearance of functional groups confirmed their participation in the adsorption process^67^.

## Conclusion

This study has investigated the efficacy of Acid Functionalized Maize Cob (AFMC) as a sustainable, cost-effective, easy, unique, efficient adosrbent ultilizing adsorption as a low-cost technique for effective removal of malachite green (cationic dye). Unique physicochemical properties of AFMC vis-à-vis high surface area (1329 m^2^/g), moisture content (12%), bulk density (0.386) enhanced the adsorptive capacity. Adsorption of malachite green onto AFMC before and after was confirmed SEM and FTIR. Effective removal of malachite green was achieved at pH 6, 10–120 min contact time, six different initial concentrations from 100 to 600 mg/L concentrations at ambient temperature. A rapid and fast kinetics was attained at 90 min with 93%% removal efficiency. Based on higher R^2^ > 0.97 and lower suitable statistical validity models (SSE, HYBRID, X^2^_,_ and MSPD), the equilibrium data were best described by Freundlich and Halsey isotherm models. The Langmuir adsorption mnolayer capacity (Qmax) of AFMC being 66.52 mg/g surpassed several adsorbents previously used for adsorption of MG. Free energy value being less than 8 kJ from DRK supported a physisorption mechanism. Based on R^2^ values and statistical error validity models, the kinetic and mechanism data were best fitted to Pseudo second order and supported by intraparticle diffusion. Subsequently, consideration could be given to AFMC as propitious material for environmental remediation.

## Supplementary Information

Supplementary Information.

## Data Availability

All data generated or analyzed during this study are included in this manuscript (and its Supplementary Information files).
