# Supplementary material for: Mechanism and isotherm modeling of effective adsorption of malachite green as endocrine disruptive dye using Acid Functionalized Maize Cob (AFMC)
Source: Sci Rep. 2021 Nov 2;11:21498. doi: 10.1038/s41598-021-00993-1 (PMC8563726; doi:10.1038/s41598-021-00993-1)
Supplement: Supplementary file 1 — Supplementary Information. [file 41598_2021_993_MOESM1_ESM.docx]

**Supplementary Document**

Mechanism and Isotherm Modeling of Effective Adsorption of Malachite Green as Endocrine Disruptive Dye using Acid Functionalized Maize Cob (AFMC)

Ojediran O. John^1,2,3^, Dada A. Oluwasogo^4,5,6*^, Aniyi O. Stephen^1,3,7^, David O. Robinson^3^, and Adewumi, A. Deborah^1,3^

^1^Landmark University SDG 7 Research Group (Grow Affordable and Clean Energy)

^2^Landmark University SDG 9 Research Group (Increase Industry, Innovation, and Infrastructure)

^3^Department of Agricultural and Biosystems Engineering, Landmark University, P.M.B.1001, Omu-Aran, Kwara State, Nigeria 9 (Increase Industry, Innovation, and Infrastructure)

^4^Landmark University SDG 6 Research Group (Clean Water and Sanitation)

^5^Landmark University SDG 11 Research Group (Sustainable Cities and Communities)

^6^Industrial Chemistry Programme, Nanotechnology Laboratory, Department of Physical Sciences, Landmark University, P.M.B.1001, Omu-Aran, Kwara, Nigeria

^7^Landmark University SDG GROUP 2 (Zero Hunger)

*Corresponding author’s e-mail: [dada.oluwasogo@lmu.edu.ng](mailto:dada.oluwasogo@lmu.edu.ng)

**S1: Preparation of Acid Functionalized Maize Cob (AFMC)**

Acid Functionalized Maize Cob (AFMC) was prepared modifying the procedure in our previous studies and in literature (Bello *et al.,* 2017; Dada *et al.,* 2017; Dada *et al.,* 2011). A carefully weighed 50 g of the screened, cleaned and carbonized maize cob was placed in a beaker containing 800 cm^3^ of 0.5 mol/dm^3^ ortho-phosphoric acid (H_3_PO_4_). The content of the beaker was thoroughly mixed and heated until a paste formed. Thereafter, it was then transferred to an evaporating dish which was placed in the furnace and heated to 300 ^o^C for thirty minutes in a muffle furnace. This was then allowed to cool and wash with distilled water to maintain a pH of 6.8 oven dried at 1000 ^o^C for six hours to constant weight and then ground. It was sieved with 106 um mesh size to obtain fine powdered of acid functionalized maize cob (AFMC) which was kept in an air-tight desiccator and used subsequently for the various experiments.

**Physicochemical Characteristics of Malachite Green**

Table S1:

Physicochemical Characteristics of Malachite Green

[CAS Number](https://en.wikipedia.org/wiki/CAS_Registry_Number): [569-64-2](http://www.commonchemistry.org/ChemicalDetail.aspx?ref=569-64-2)

IUPAC Name: 4-{[4-(Dimethylamino) phenyl](phenyl)methylidene}-*N*,*N*-dimethylcyclohexa-2,5-dien-1-iminium Chloride

Dye Classification: Cationic Dye

Molecular formular: C_23_H_25_ClN_2_ (chloride)

Molar Mass: 364.911 g/mol

Max. Wavelength: 617 nm

Melting Point 164 ^o^C

Solubility in Water 40 g/L at 25°C

Other names: Aniline green; Basic green 4; Diamond green B; Victoria green B

Color index number: 42000

**Some physico-chemical parameters of the AFMC**

**Table S2: Some physico-chemical parameters of the FZMC**

| Properties | AFMC |
| --- | --- |
| Surface Area (m^2^/g) | 1329 |
| pH | 6.75 |
| % Moisture Content | 12 |
| % Loss of mass on ignition | 0.9 |
| Bulk density (g/cm^3^) | 0.386 |
| Particle size | 106 μm |

**Figure S1, S2, S3 Plot of Effect of Operational Parameters (pH, initial concentration and contact time) at optimum condition**

Figure S1. Effect of pH on adsorption of MG dye onto AFMC

*Experimental conditions: MG dye Concentration = 100 mg/L; Volume of MG solution = 50 mL;*

*Adsorbent dose = 100 mg; stirring speed = 200 rpm, contact time = 90 min*.

Figure S2: Effect of initial concentration on adsorption of MG onto AFMC

*Experimental conditions: Volume of MG solution = 50 mL; AFMC dose = 100 mg;*

*pH = 6.0, contact time = 90 min, and temperature = 25± 2 ^o^C.*

Figure. S3: Effect on contact time on adsorption of MG onto AFMC

*Experimental conditions: Volume of MG solution = 50 mL; AFMC dose = 100 mg;*

*pH = 6.0, and temperature = 25± 2 ^o^C*
